# L1CAM expression in either metastatic brain lesion or peripheral blood is correlated with peripheral platelet count in patients with brain metastases from lung cancer

**DOI:** 10.3389/fonc.2022.990762

**Published:** 2022-10-27

**Authors:** Jia-Wei Wang, Hong-Liang Wang, Qi Liu, Ke Hu, Qing Yuan, Sheng-Kai Huang, Jing-Hai Wan

**Affiliations:** ^1^Department of Neurosurgery, National Cancer Center/National Clinical Research Center for Cancer/Cancer Hospital, Chinese Academy of Medical Sciences and Peking Union Medical College, Beijing, China; ^2^Department of Neurosurgery, The Second Affiliated Hospital of Anhui Medical University, Hefei, China; ^3^Department of Clinical Laboratory, National Cancer Center/National Clinical Research Center for Cancer/Cancer Hospital, Chinese Academy of Medical Sciences and Peking Union Medical College, Beijing, China

**Keywords:** L1 cell adhesion molecule, brain metastasis, lung cancer, systemic immune-inflammation state, platelet

## Abstract

**Background:**

Systemic immune-inflammation states across the heterogeneous population of brain metastases from lung cancer are very important, especially in the context of complex brain-immune bidirectional communication. Previous studies from our team and others have shown that the L1 cell adhesion molecule (L1CAM) is deeply involved in the aggressive phenotype, immunosuppressive tumor microenvironment (TME), and metastasis during multiple malignancies, which may lead to an unfavorable outcome. However, little is known about the relationship between the L1CAM expression and the systemic immune-inflammation macroenvironment beyond the TME in brain metastases from lung cancer.

**Methods:**

Two cohorts of patients with brain metastases from lung cancer admitted to the National Cancer Center, Cancer Hospital of Chinese Academy of Medical Sciences, were studied in the present research. The L1CAM expression in cranial metastatic lesions by immunohistochemistry was explored in patients treated with neurosurgical resection, whereas the L1CAM expression in peripheral blood by ELISA was tested in patients treated with non-surgical antitumor management. Furthermore, based on peripheral blood cell counts in the CBC test, six systemic immune-inflammation biomarkers [neutrophil count, lymphocyte count, platelet count, systemic immune-inflammation index (SII), neutrophil-to-lymphocyte ratio (NLR), and platelet-to-lymphocyte ratio] were calculated. Then, the relationship between the L1CAM expression and these systemic immune-inflammation biomarkers was analyzed. In addition, these systemic immune-inflammation biomarkers were also used to compare the systemic immune-inflammation states in two cohorts of patients with brain metastases from lung cancer.

**Results:**

Positive L1CAM expressions in the metastatic brain lesions were accompanied with significantly increased peripheral platelet counts in patients treated with neurosurgical tumor resection (*P* < 0.05). Similarly, in patients treated with non-surgical antitumor management, L1CAM expressions in the peripheral blood were positively correlated with peripheral platelet counts (*P* < 0.05). In addition, patients prepared for neurosurgical tumor resection were presented with poorer systemic immune-inflammation states in comparison with the one with non-surgical antitumor management, which was characterized by a significant increase in peripheral neutrophil counts (*P* < 0.01), SII (*P* < 0.05), and NLR (*P* < 0.05) levels.

**Conclusion:**

The L1CAM expression in either the metastatic brain lesion or peripheral blood is positively correlated with the peripheral platelet count in patients with brain metastases from lung cancer. In addition, brain metastases that are prepared for neurosurgical tumor resection show poor systemic immune-inflammation states.

## Introduction

As a hallmark of cancer, systemic immune-inflammation perturbation has been extensively explored in the clinical and preclinical scenarios involving peripheral cancer (1–4). When it comes to cancer of the central nervous system (CNS), the well-known bidirectional interaction between the CNS and the immune system leads to a more complex modulation of the systemic immune-inflammation landscape in the host with CNS cancer (5–7). Moreover, recent studies have indicated that the systemic immune-inflammation perturbation is not unique to a specific malignant brain tumor itself but rather occurs in response to brain injury, which means that the extent of CNS injury may influence the extent of systemic immune-inflammation perturbation (8). In the therapeutic strategies of brain metastases from lung cancer, neurosurgical tumor resection is always indicated in the treatment for patients with symptomatic, large, or accessible solitary lesion or, in certain circumstances, when there is a single large lesion that is life-threatening or produces a mass effect among multiple lesions (9–12). Currently, the systemic immune-inflammation landscape comparing the one indicated for neurosurgical tumor resection and that treated with other non-surgical antitumor management is relatively limited in brain metastasis from lung cancer.

As a transmembrane glycoprotein of the immunoglobulin superfamily, the L1 cell adhesion molecule (L1CAM) plays an essential role in the development of the CNS, but it is also highly relevant to the malignant human tumors (13, 14). Vast evidence has supported the fact that the L1CAM is a major driver for tumor cell invasion and motility (15). Recently, our team has demonstrated that brain metastases from lung cancer show an aberrant expression of the L1CAM and that the L1CAM is an independent predictive factor of poor prognosis for brain metastases from lung cancer (16). Furthermore, the extracellular domain of the L1CAM can be a regulatory membrane proximally cleaved from the tumor cell surface or tumor cell–derived vesicles by several proteases such as the disintegrin and metalloprotease (ADAM) family (15, 17). The shedding and water-soluble extracellular domain is able to mediate L1CAM signal transduction *via* homo- and heterophilic interactions similarly. To date, L1CAM research in the cancer area has focused heavily on local responses in the tumor microenvironment (TME); the relationship between the L1CAM and systemic immune-inflammation landscape beyond the TME remains to be fully determined, especially in the patients with brain metastases from lung cancer.

Considering the accessibility, convenience, and cost–benefit analysis among the assessing methods, biomarkers based on the complete blood count (CBC) test of peripheral blood are widely used in clinics and labs to reflect the systemic immune-inflammation state (18, 19). The parameters retracted from the CBC test mainly involve peripheral neutrophil, lymphocyte, and platelet counts and three blood cell ratios, namely the systemic immune-inflammation index (SII), the neutrophil-to-lymphocyte ratio (NLR), and the platelet-to-lymphocyte ratio (PLR) (1, 2, 20–22). In the present study, we firstly tested the L1CAM expression in the metastatic brain lesion in patients with brain metastases from lung cancer who were treated with neurosurgical tumor resection and the L1CAM expression in peripheral blood in the one treated with non-surgical antitumor management. Secondly, the relationship between the L1CAM expression and biomarkers mapping the systemic immune-inflammation state in patients was analyzed. Finally, the biomarkers based on the CBC test in the patients treated with neurosurgical tumor resection or treated with non-surgical antitumor management were compared to depict the extent of systemic immune-inflammation perturbations in the two kinds of patients.

## Materials and methods

This research was approved by the Ethics Committee of the Cancer Hospital, Chinese Academy of Medical Sciences (No. 22/052-3253), and was performed in accordance with the World Medical Association Declaration of Helsinki. Informed consents were obtained from all participants.

### Study cohort 1: patients treated with neurosurgical tumor resection

Metastatic cranial tumor tissues of neurosurgically resected brain metastases from lung cancer between March 2014 and October 2019 were obtained from a tissue bank in the Department of Neurosurgery at the National Cancer Center (NCC), Cancer Hospital of the Chinese Academy of Medical Sciences, and used for the construction of tissue microarrays (TMAs).

The TMA construction and subsequent immunohistochemistry (IHC) of the L1CAM in TMA sections have been described previously with details by our team (16). The IHC evaluation of the L1CAM expression in metastatic brain lesions was independently performed by two experienced investigators who were blinded to patients’ demographics, with discrepancies resolved by consensus under a microscope for multiviewing.

### Study cohort 2: patients treated with non-surgical antitumor management

Remanent outpatient venous serum samples after a blood test from patients with brain metastases from lung cancer who were treated with non-surgical antitumor management in NCC, Cancer Hospital of the Chinese Academy of Medical Sciences, between November 2021 and December 2021 were collected and further tested for the soluble L1CAM in peripheral blood by enzyme-linked immunosorbent assay (ELISA).

A commercially available solid-phase sandwich ELISA kit was used to detect and quantify the level of the human L1CAM in the serum samples according to the manufacturer’s protocol (Invitrogen, Thermo Fisher Scientific, USA). In brief, human-L1CAM antibody-coated 96-well plates were incubated with serum samples. After washing, the secondary antibody (biotin conjugate anti-human L1CAM) was added, followed by streptavidin-horseradish peroxidase (HRP) solution and TMB substrate. The reaction was then stopped with a stop solution. The plates were washed between each of the previous steps with a wash buffer. ELISAs were run in duplicates, and the 96-well plates were read at 450 nm on a microtiter plate reader. L1CAM concentrations were then determined using a standard curve as detailed by the manufacturer’s guidelines.

### Participant demographics and biomarkers based on the CBC test in two cohorts

Data concerning the clinical/pathological parameters of each participant were retracted from their medical records, including age/gender of patients, Karnofsky Performance Status (KPS) scores, locations and numbers of brain metastases, with or without extracranial transfer, pathology subtype, gene alterations, CBC test sheet, and treatment information.

In study cohort 1, peripheral neutrophil, lymphocyte, and platelet counts before neurosurgical resection were recorded. Furthermore, in study cohort 2, peripheral neutrophil, lymphocyte, and platelet counts were recorded from the blood samples that were synchronously tested for subsequent ELISA. Moreover, three systemic immune-inflammation biomarkers (SII, NLR, and PLR) were then calculated based on the previously neutrophil, lymphocyte, and platelet counts in the CBC test sheet; calculation formulas were as follows: SII = (neutrophils * platelets)/lymphocytes, NLR = neutrophils/lymphocytes, and PLR = platelets/lymphocytes.

### Statistical analysis

Prism version 9.0 (GraphPad software, San Diego, CA,USA) and SPSS Statistics software version 26 (SPSS, Inc., Chicago, IL, USA) were used for the statistical analysis and graphing. The data were presented as number, percentage, or mean ± S.D. In study cohort 1, the levels of biomarkers based on the CBC test in the L1CAM-negative and L1CAM-positive patients tested by IHC were compared using unpaired t test. In study cohort 2, the correlation between the levels of biomarkers based on the CBC test and the L1CAM expression in blood by ELISA test was analyzed with Pearson correlation. The unpaired t test and χ^2^ test were used to assess the clinic-pathological parameters in two cohorts. Statistical significance was accepted with P < 0.05.

## Results

### L1CAM expression by IHC in brain metastases and CBC biomarkers

**Figure 1:**
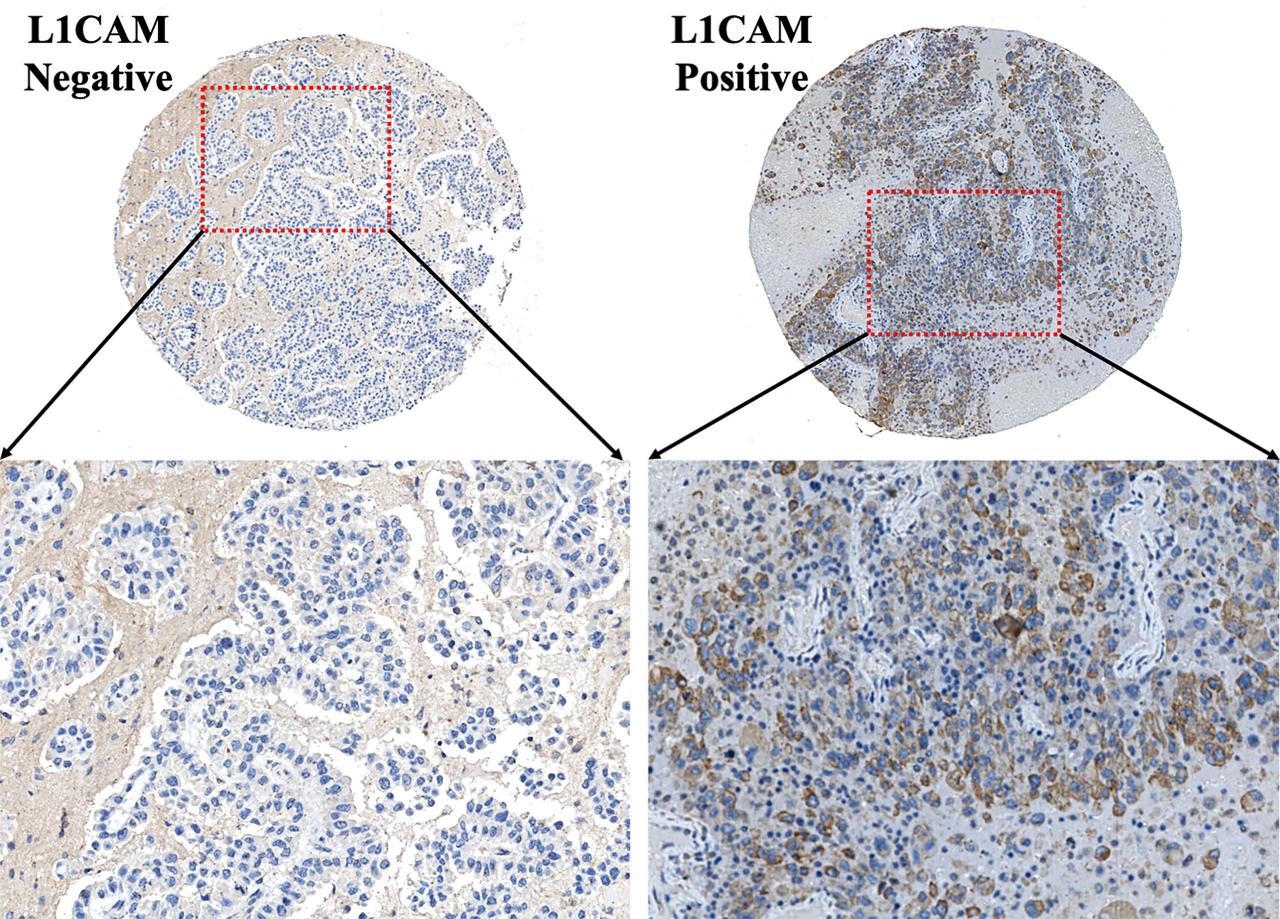
Typical IHC images of the L1CAM on the TMA section of brain metastases from lung cancer. L1CAM-positive tumor cells showed significant brown staining of cell membranes. L1CAM, L1 cell adhesion molecule; IHC, immunohistochemistry; TMA, tissue microarray.

**Figure 2:**
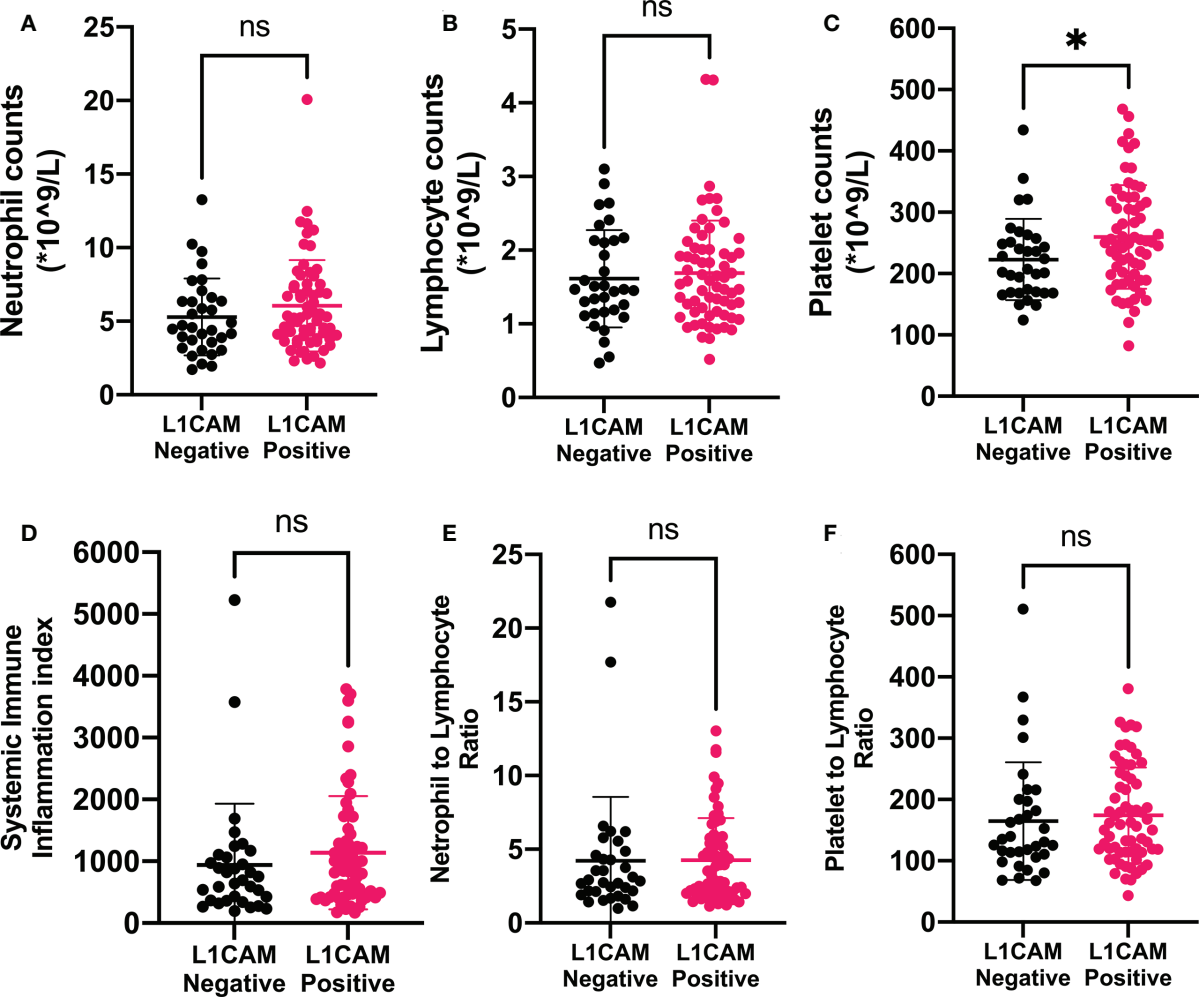
Associations of immune-inflammation biomarkers and L1CAM expression in metastatic brain lesion in patients treated with neurosurgical tumor resection. Data showed that positive L1CAM expressions in the metastatic brain lesions were accompanied by significantly increased peripheral platelet counts **(C)**. L1CAM, L1 cell adhesion molecule; SII, systemic immune-inflammation index; NLR, neutrophil-to-lymphocyte ratio; PLR, platelet-to-lymphocyte ratio. *P < 0.05, ^ns^*P* > 0.05.

In the present study, 98 patients with brain metastases from lung cancer who received neurosurgical tumor resection in our institute were included in the final analysis to explore the relationship between the L1CAM expression in brain metastases and CBC biomarkers. The overview of the demographics of these patients (cohort 1) is shown in **Table 1**. Sixty-five (66.33%) among these 98 patients showed a positive L1CAM expression in metastatic brain lesions. As indicated in **Figure 1**, positive L1CAM sections were presented with the staining of cell membranes, especially in the tumor–stroma interface. We further divided the patients into the L1CAM-negative group and the L1CAM-positive group (**Figure 2**). Moreover, our study indicated that the patients with a L1CAM-positive expression had a significant increase in the platelet count of peripheral blood compared with the one with a L1CAM-negative expression (P < 0.05, 259.6 ± 84.43 *vs*. 222.9 ± 66.15 * 10^9^/l). There were no significant differences in other CBC biomarkers including the neutrophil count, lymphocyte count, SII, NLR, and PLR between the L1CAM-negative group and the L1CAM-positive group (P > 0.05).

**Table 1:** Demographics of the two cohorts of patients with brain metastases from lung cancer.

Clinical parameters	Brain metastasis from lung cancer		*P*
	Cohort 1 (n = 98)	Cohort 2 (n = 40)	
Age (years)	58.64 ± 10.91	57.48 ± 10.42	0.564
Gender			
Male	53 (54.08%)	17 (42.50%)	0.217
Female	45 (45.92%)	23 (57.50%)
KPS scores	93.06 ± 13.65	97.00 ± 6.87	0.085
Pathological type			
AC	80 (81.63%)	28 (70.00%)	0.074
SCC	1 (1.02%)	4 (10.00%)
SCLC	8 (8.16%)	4 (10.00%)
Others	9 (9.18%)	4 (10.00%)
BM locations			
Supra. dominant	79 (80.61%)	37 (92.50%)	0.083
Infra. dominant	19 (19.39%)	3 (7.50%)
BM number			
Single	49 (50.00%)	12 (30.00%)	*0.032*
Multiple	49 (50.00%)	28 (70.00%)
Extracranial transfer			
No	75 (76.53%)	13 (32.50%)	*0.000*
Yes	23 (23.47%)	27 (67.50%)
Gene alterations			
EGFR mutation	29 (29.59%)	24 (60.00%)	*0.000*
ALK rearrangement	3 (3.06%)	5 (12.50%)
Neg. or unknow	66 (67.35%)	11 (27.50%)
Treatment history			
Naïve	63 (64.29%)	0 (0%)	*0.000*
Systemic therapy* only	16 (16.33%)	23 (57.50%)
Radiotherapy** only	6 (6.12%)	0 (0%)
Both systemic or radiotherapy	13 (13.27%)	17 (42.50%)

L1CAM, L1 cell adhesion molecule; KPS, Karnofsky Performance Status; AC, adenocarcinoma; SCC, squamous cell carcinoma; SCLC, small cell lung cancer; BM, brain metastasis; Supra., supratentorial; Infra., infratentorial; Neg. or unknow, negative or unknown; EGFR, epidermal growth factor receptor; ALK, anaplastic lymphoma kinase. *: including chemotherapy, molecularly targeted therapy and immunotherapy. **: including stereotactic radiosurgery and whole brain radiation therapy.

The bold values indicated significant differences.

### L1CAM expression by ELISA in peripheral blood and CBC biomarkers

In the present study, 40 patients with brain metastases from lung cancer who received non-surgical antitumor management in our institute were included to explore the relationship between the L1CAM expression in peripheral blood and CBC biomarkers. The overview of demographics of these patients (cohort 2) is also shown in **Table 1**. The mean (± SD) concentration of the L1CAM in the serum of these patients detected by ELISA was 1,940.76 ± 470.30 pg/ml. Pearson’s correlation analysis revealed that the L1CAM concentration in peripheral blood had a significantly positive correlation with the platelet count of peripheral blood (P = 0.0178, r = 0.3729, 95% CI: 0.06945 to 0.6132, **Table 2**). Moreover, other CBC biomarkers including the neutrophil count, lymphocyte count, SII, NLR, and PLR did not show a significant association with the L1CAM concentration in the cohort 2 group of patients (P > 0.05).

**Table 2:** Pearson correlation of L1CAM expression in peripheral blood with biomarkers indicating systemic immune-inflammation states.

Pearson correlation	L1CAM in peripheral blood					
	Neutrophil	Lymphocyte	Platelet	SII	NLR	PLR
r	0.02914	0.1395	0.3729	0.02862	-0.1797	0.1258
95% CI	-0.2850 to 0.3376	-0.1798 to 0.4322	0.06945 to 0.6132	-0.2854 to 0.3371	-0.4652 to 0.1396	-0.1933 to 0.4208
R squared	0.0008492	0.01947	0.1391	0.0008190	0.03229	0.01582
*P value*	0.8583	0.3906	*0.0178*	0.8608	0.2672	0.4393

L1CAM, L1 cell adhesion molecule; SII, systemic immune-inflammation index; NLR, neutrophil-to-lymphocyte ratio; PLR, platelet-to-lymphocyte ratio; 95% CI, 95% confidence interval.

The bold values indicated significant differences.

### CBC biomarkers in the two cohorts of brain metastases from lung cancer

**Figure 3:**
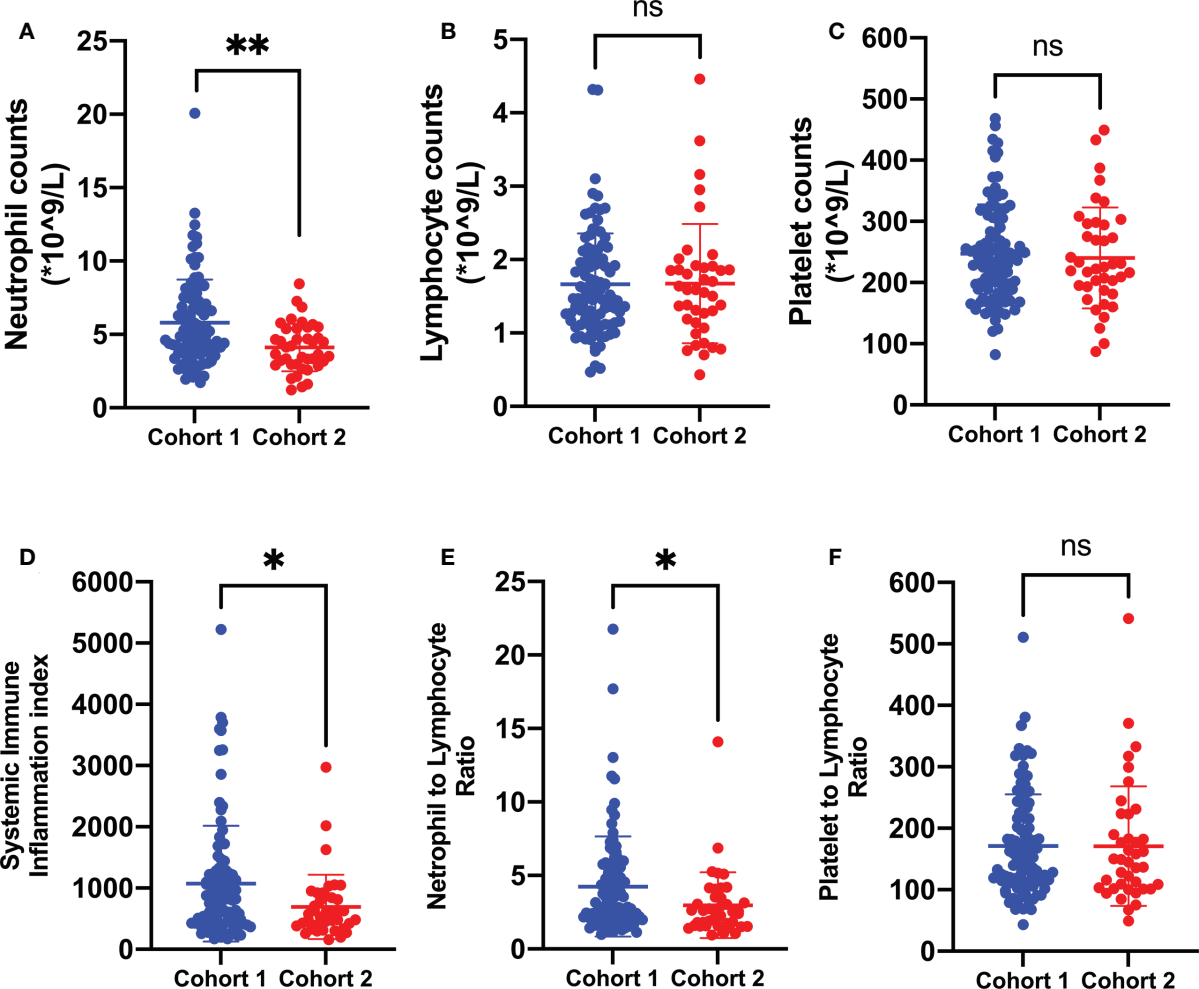
Biomarkers of systemic immune-inflammation states in the two cohorts of brain metastases from lung cancer. In comparison with the one treated with non-surgical antitumor management, patients prepared for neurosurgical tumor resection were presented with poorer systemic immune-inflammation states, which was characterized by a significant increase in peripheral neutrophil counts **(A)**, SII **(D)**, and NLR **(E)** levels. Cohort 1: patients treated with neurosurgical tumor resection; cohort 2: patients treated with non-surgical antitumor management. SII, systemic immune-inflammation index; NLR, neutrophil-to-lymphocyte ratio; PLR, platelet-to-lymphocyte ratio. **P < 0.01, *P < 0.05, ^ns^*P* > 0.05.

As indicated in **Table 1**, there were no significant differences in terms of age, gender, KPS scores, pathological subtype, and locations of brain metastases between the two cohorts (P > 0.05). The patients with a single metastatic lesion constituted half of the cohort 1 group while only 30% of the cohort 2 group. Moreover, the presence of extracranial transfer was more frequent in the cohort 2 group (67.50%) than in the cohort 1 group (23.47%). In addition, the gene alterations and treatment history also showed significant differences in the two cohorts. In order to explore the systemic immune-inflammation states in the two cohorts of brain metastases, CBC biomarkers were further analyzed and compared. As shown in **Figure 3**, the patients who were prepared for neurosurgical tumor resection had significantly systemic immune-inflammation perturbation, which was characterized by a significant increase in the neutrophil count (P < 0.01, 5.79 ± 2.96 *vs*. 4.11 ± 1.62*10^9^/l), SII (P < 0.05, 1,072.28 ± 943.00 *vs*. 690.43 ± 525.41), and NLR levels (P < 0.05, 4.25 ± 3.40 *vs*. 2.98 ± 2.24) in comparison with the one receiving non-surgical antitumor management.

## Discussion

In the present study, the L1CAM expression in the metastatic brain lesions or peripheral blood and biomarkers mapping the systemic immune-inflammation state was investigated in patients with brain metastases from lung cancer. The main findings are as follows: (1) Positive L1CAM expressions in the metastatic brain lesions were accompanied by significantly increased peripheral platelet counts in patients treated with neurosurgical tumor resection. (2) In patients treated with non-surgical antitumor management, L1CAM expression in the peripheral blood was positively correlated with peripheral platelet counts. (3) Patients prepared for neurosurgical tumor resection were presented with poorer systemic immune-inflammation states, which was characterized by a significant increase in peripheral neutrophil counts and SII and NLR levels. These findings suggest a relationship between the platelet, L1CAM expression, and tumor brain metastasis and remind us that we should pay special attention to the systemic immune-inflammation perturbations in patients treated with neurosurgical tumor resection.

In the past decades, mounting evidence from preclinical and clinical studies has supported the role of platelets in tumorigenesis and metastasis involving a wide variety of complex, bidirectional cross-talks between platelets and cancer cells in the blood and TME (23–25). However, the mechanisms underlying the intercommunication remain to be fully determined. Recently, it has been demonstrated by our team and others that the transmembrane glycoprotein L1CAM is overexpressed in multiple human malignancies brain metastases (16), glioma (26, 27), non-small cell lung cancer (28), and colon cancer (14). Furthermore, L1CAM overexpression in these cancer contexts is generally associated with poor prognosis (26, 29–31), an invasive phenotype (32, 33), advanced tumor stages (28, 34), and chemotherapy resistance (35). In addition, the L1CAM in the tumor cell surface or the shedding L1CAM cleaved from the tumor cell surface into blood can exert their biological function mainly through binding to themselves (homophilic) or other partners such as integrins (heterophilic). Our study found that the L1CAM expression in either metastatic brain lesion or peripheral blood is positively correlated with the peripheral platelet count in patients with brain metastases from lung cancer. To the best of our knowledge, our study firstly provided *in vivo* evidence for the potential bridge effect of the L1CAM in the cross-talk between the platelet and cancer.

Emerging evidence indicates that the synchronous expression of the L1CAM and platelet count in cancer and peripheral blood may lead to a plethora of interactions between cancer and platelet, which will add to the functional significance of this cross-talk. For example, in the cancer setting, tumor-activated platelets may release a large amount of transforming growth factor (TGF)-β1 (36, 37), which can strongly upregulate the L1CAM expression in multiple cell lines (38–40). Then, the upregulation of the L1CAM further triggers its binding to partner integrins, resulting in an amplification of the overlapping previous TGF-β1-integrin signaling. Finally, cumulative TGF-β1–L1CAM–integrin signaling leads to nuclear factor (NF)-kappaB activation (15, 32). Reciprocally, previous studies have also indicated that inhibition of TGF-β1 signaling or L1CAM signaling abrogated these effects and likewise inhibited platelet-derived TGF-β1/L1CAM/integrin/NF-kappaB pathways induced tumor invasion metastasis cascade (23, 25, 33, 40). In addition, since platelets express integrins while vascular endothelial cells also express the L1CAM (41), and L1CAM–integrin interactions can occur in trans(between different cells) (15), it is feasible that L1CAM–integrin signaling will contribute to significant platelet adhesion and tumor-associated thrombosis, which is thought to be important for the tumor cell arrest and migration in blood circulation when tumor cells detach from the primary site and intravasate into blood vessels (42, 43). Taken together, these studies suggest that the L1CAM plays a crucial role in the bidirectional cross-talk between cancer and platelets.

In the present study, patients in cohort 2 treated with the non-neurosurgical antitumor management presented with more brain metastatic lesions, higher incidence of extracranial transfer, and more complex treatment history in comparison with patients in cohort 2 who were prepared for the neurosurgical tumor resection. All of these clinical parameters may result in the perturbations in systemic immune-inflammation states in clinics (1, 2, 4, 21, 44, 45). Generally, more brain metastatic lesions, higher incidence of extracranial transfer, and more complex treatment history are usually associated with poor prognosis and poorer systemic immune-inflammation states in brain metastasis from lung cancer (3, 9, 11, 46–48). However, that is not the case in our study. Our study indicated that patients prepared for neurosurgical tumor resection had poorer systemic immune-inflammation states. In the setting of brain metastases from lung cancer, neurosurgical tumor resection and non-neurosurgical antitumor management including chemotherapy and radiation are the mainstays of the therapeutic strategies (9, 11, 47). Furthermore, in our institution, neurosurgical resection is always indicated in the symptomatic, large, or accessible solitary lesions or, in certain circumstances, when there is a single large lesion that is life-threatening or produces a mass effect among multiple lesions; on the other hand, the non-neurosurgical antitumor management is used in the context of lack of significant intracranial hypertension due to brain metastases. Thus, we speculated that the intracranial mass effect resulting from the intra-axial metastatic brain lesions and peritumoral edema may rank at the top of the hierarchy of all clinical parameters affecting the systemic immune-inflammation states; further studies with large samples are needed to address this issue. This knowledge should be integrated into the treatment strategies in the context of CNS cancer in order to improve the outcome.

Considering the predictive role of the L1CAM and systemic immune-inflammation biomarkers based on the CBC test in the therapeutic efficacy and prognosis in the context of multiple cancers such as lung cancer (28, 34), colorectal cancer (49, 50), and brain metastases (16, 27, 51), the dynamic quantification of the L1CAM in serum and systemic immune-inflammation biomarkers may act as potentially predictive biomarkers for cancer therapy such as radiation and help to guide patient stratification and treatment decisions, especially when accumulating evidence has shown that both the L1CAM and its strong agonist TGF-β can be induced by radiation (1, 52, 53). We know that the impact of radiation on the immune system is highly heterogenous, which can induce remodeling of systemic immunity either impeding or augmenting overall treatment efficacy and make it challenging to understand the assembling impact on the systemic immune. Our findings from peripheral blood may provide a promising assessing method with the accessibility and convenience across these contexts. In addition, our findings may also expand on the implications for where the metastasis has expanded and implications for other types of cancer (54, 55), since previous studies have shown that the L1CAM is expressed in other types of cancer such as breast cancer, which is also a major primary lesion of brain metastases, and the L1CAM promotes breast cancer cell adhesion and migration *in vitro (* 56, 57). Further studies in the future are needed to address this issue.

It should be noted that there are some limitations in the present study. Firstly, in the present study, the L1CAM expression in metastatic brain lesion was investigated in patients prepared for neurosurgical resection, whereas the L1CAM expression in the peripheral blood was tested in another cohort treated with non-neurosurgical antitumor management. In our institution, the tumor samples of brain metastases rather than blood samples are commonly collected and stored in the tissue bank. Since our present study in cohort 1 is a retrospective study, we lack the presurgical peripheral blood sample of cohort 1. Thus, the soluble L1CAM in peripheral blood data in study cohort 1 was not tested in the present study. We believe that it would be better if the soluble L1CAM in peripheral blood was performed in both cohorts; especially, adding pre- and postoperative data of the soluble L1CAM in the peripheral blood in study cohort 1 would be more convincing. Actually, prospective studies involving the dynamic L1CAM expression in both pre- and postoperative states following different treatment modalities have been started in our team. Secondly, our study indicates that the L1CAM expression in both metastatic brain lesion and peripheral blood is associated with the peripheral platelet count. However, the mechanisms underlying this correlation and the subsequent biological function of this correlation are unknown and not explored in the present study. Further studies have been carried out in our institution to investigate the relationship between platelet–L1CAM signaling, systemic immune macroenvironment, TME, and cancer biological behaviors. Thirdly, in the present study, six biomarkers based on CBC are analyzed to depict the systemic immune-inflammation states in the two cohorts of brain metastases, and there are significant differences in both cohorts. Our findings cannot exclude the fact that there may be several other biomarkers with more sensitivity and specificity that can map the systemic immune-inflammation states.

In conclusion, the L1CAM expression in either metastatic brain lesion or peripheral blood is positively correlated with the peripheral platelet count in patients with brain metastases from lung cancer. In addition, brain metastases that are prepared for neurosurgical tumor resection show poorer systemic immune-inflammation states.

## Data availability statement

The raw data supporting the conclusions of this article will be made available by the authors, without undue reservation.

## Ethics statement

This study was reviewed and approved by Ethics Committee of the Cancer Hospital, Chinese Academy of Medical Sciences (No.22/052-3253). The patients/participants provided their written informed consent to participate in this study.

## Author contributions

QY, S-KH and J-HW conceived of and designed the study. J-WW, H-LW, KH and QL performed the data collection and statistical analyses. J-WW drafted the initial manuscript. H-LW,QY, S-KH, and J-HW made critical comments and revision for the initial manuscript. All authors contributed to the article and approved the submitted version.

## Funding

This work was supported by grants from the Beijing Xisike Clinical Oncology Research Foundation (Y-QL202101-0094), National Natural Science Foundation of China (82072803), and CAMS Innovation Fund for Medical Sciences (2021-I2M-1-012).

## Conflict of interest

The authors declare that the research was conducted in the absence of any commercial or financial relationships that could be construed as a potential conflict of interest.

## Publisher’s note

All claims expressed in this article are solely those of the authors and do not necessarily represent those of their affiliated organizations, or those of the publisher, the editors and the reviewers. Any product that may be evaluated in this article, or claim that may be made by its manufacturer, is not guaranteed or endorsed by the publisher.
